# Neurocognitive function as outcome and predictor for prefrontal transcranial direct current stimulation in major depressive disorder: an analysis from the DepressionDC trial

**DOI:** 10.1007/s00406-024-01759-2

**Published:** 2024-02-26

**Authors:** Aldo Soldini, Ulrike Vogelmann, Sabine Aust, Stephan Goerigk, Christian Plewnia, Andreas Fallgatter, Claus Normann, Lukas Frase, Peter Zwanzger, Thomas Kammer, Carlos Schönfeldt-Lecuona, Gizem Vural, Malek Bajbouj, Frank Padberg, Gerrit Burkhardt

**Affiliations:** 1 Department of Psychiatry and Psychotherapy, LMU University Hospital, Munich, Germany; 2 International Max Planck Research School for Translational Psychiatry, Munich, Germany; 3 Department of Psychiatry and Neurosciences, Charité - Universitätsmedizin Berlin, Berlin, Germany; 4 Charlotte Fresenius Hochschule, Munich, Germany; 5 Department of Psychiatry and Psychotherapy, German Center for Mental Health (DZPG), Tübingen Center for Mental Health, University of Tübingen, Tübingen, Germany; 6 Department of Psychiatry and Psychotherapy, University of Freiburg, Breisgau, Germany; 7 Center for Basics in Neuromodulation, University of Freiburg, Freiburg, Germany; 8 Department of Psychosomatic Medicine and Psychotherapy, University of Freiburg, Breisgau, Germany; 9 Clinical Center for Psychiatry, Psychotherapy, Psychosomatic Medicine, Geriatrics and Neurology, Kbo-Inn-Salzach-Klinikum, Gabersee, Germany; 10 Department of Psychiatry and Psychotherapy III, University of Ulm, Ulm, Germany

**Keywords:** Transcranial direct current stimulation, Non-invasive brain stimulation, Major depressive disorder, Depression, Cognition, Neurocognitive tests

## Abstract

Transcranial direct current stimulation (tDCS) of the prefrontal cortex might beneficially influence neurocognitive dysfunctions associated with major depressive disorder (MDD). However, previous studies of neurocognitive effects of tDCS have been inconclusive**.** In the current study, we analyzed longitudinal, neurocognitive data from 101 participants of a randomized controlled multicenter trial (DepressionDC), investigating the efficacy of bifrontal tDCS (2 mA, 30 min/d, for 6 weeks) in patients with MDD and insufficient response to selective serotonin reuptake inhibitors (SSRI). We assessed whether active tDCS compared to sham tDCS elicited beneficial effects across the domains of memory span, working memory, selective attention, sustained attention, executive process, and processing speed, assessed with a validated, digital test battery. Additionally, we explored whether baseline cognitive performance, as a proxy of fronto-parietal-network functioning, predicts the antidepressant effects of active tDCS versus sham tDCS. We found no statistically significant group differences in the change of neurocognitive performance between active and sham tDCS. Furthermore, baseline cognitive performance did not predict the clinical response to tDCS. Our findings indicate no advantage in neurocognition due to active tDCS in MDD. Additional research is required to systematically investigate the effects of tDCS protocols on neurocognitive performance in patients with MDD.

**Supplementary Information:**

The online version contains supplementary material available at 10.1007/s00406-024-01759-2.

## Introduction

Transcranial Direct Current Stimulation (tDCS) is a form of non-invasive brain stimulation (NIBS) that utilizes electrodes on the scalp to create a weak electrical current in order to modulate cortical excitability [1]. In the treatment of major depressive disorder (MDD), anodal tDCS is usually applied over the left dorsolateral prefrontal cortex (DLPFC) [2], a brain area which contributes to frontoparietal network (FPN) function [3]. The FPN plays a central role for several cognitive domains, like attention[4], working memory [5], memory span [6] executive function [7], processing speed[8], and cognitive control [9]. Poor performance in these cognitive domains has also been associated with depressive disorders [10–14]. Therefore, it seems plausible that stimulation of the FPN could influence performance in these domains and that baseline cognitive performance, as a proxy of FPN functioning, could predict the clinical effects of stimulation.

Previous studies have investigated the neurocognitive effects of tDCS when applied to the DLPFC in patients with MDD reporting significant time-dependent improvements in attention/vigilance, working memory, executive functioning, processing speed, and social cognition when compared to placebo [15–18]. On the other hand, multiple studies report no statistically significant group-by-time interaction effects [19–27]. A recent meta-analysis of the cognitive effects of tDCS across multiple disorders revealed that active tDCS elicited improvements in attention/vigilance, and working memory when compared to sham tDCS [28]. This meta-analysis was based on studies that were very heterogeneous in designs, sample sizes, outcomes, and main findings. Thus, a study with a large sample size would be warranted to further test the effects of tDCS on cognition in patients with MDD. To the best of our knowledge, no studies have investigated baseline cognitive testing as a predictor of affective response to tDCS.

In this ancillary analysis of a triple-blind, randomized, sham-controlled multicenter trial, we investigated whether a standard bifrontal tDCS protocol compared to sham tDCS alters cognitive performance across the domains of memory span, working memory, selective attention, sustained attention, executive functioning, and processing speed. Additionally, we explored whether baseline cognitive performance as a proxy of FPN functioning predicts the antidepressant effects of tDCS versus sham tDCS.

## Methods and materials

### Study population

We analyzed data from the DepressionDC trial (trial registration number: NCT02530164); a triple-blind, randomized, sham-controlled clinical trial carried out across eight psychiatric centers in Germany [29]. The study investigated the efficacy and safety of tDCS as a treatment for MDD in patients that did not respond to conventional pharmacological treatment with selective serotonin reuptake inhibitors (SSRIs). Patients were originally randomized to receive 24 sessions within 6 weeks of either active or sham tDCS. The montage employed in tDCS involves placing the anode over F3 and the cathode over F4. Active stimulation consisted of a constant 2 mA direct current that lasted for 30 min. The sham paradigm consisted of a ramp-up and ramp-down sequence to induce similar skin sensations as active tDCS. tDCS was applied using a DC-stimulator (‘Mobile’, neuroConn GmbH, Ilmenau, Germany). Inclusion and exclusion criteria are reported in the supplement. Local ethics committees approved the study at each study site. All participants gave their written informed consent before inclusion in the study. From an initial sample total of 150 patients (intention-to-treat sample)**,** we analyzed the data from 101 patients that had available neuropsychological assessments. Data from 49 patients were missing due to technical errors, organizational difficulties at the local treatment sites, and refusal to participate.

### Neurocognitive test battery

Neurocognitive function was assessed longitudinally during the study at baseline, post-treatment (week 6), and at the 6-month follow-up using the EmoCogMeter, a digitalized, validated cognitive test battery developed at the Charite Berlin [30–32]. The EmoCogMeter examines the domains of memory span, working memory, selective attention, sustained attention, executive function, and processing speed. Memory span is tested by a digit-span assessment [33]. Working memory was assessed by an n-back task [13]. A variant of the Stroop test and a working memory component were used to assess selective attention and sustained attention, respectively [34]. executive function was measured by both the Trail Making B [35] and Tower of Hanoi tests [36]. Finally, processing speed was measured using a symbol letter modalities test, a variation of the symbol digit modality test. For additional technical information about the tests, please refer to the supplement.

### Further outcome measures

The severity of the depressive episode was assessed by trained clinical staff utilizing the Montgomery-Åsberg Depression Rating Scale (MADRS), which was also chosen for the primary outcome of the study [37]. Severity is classified as an absence of symptoms (0–6 points), mild depressive episode (7–19 points), moderate depressive episode (20–34 points), or severe depressive episode (35–60 points). State and trait anxiety were measured utilizing The State-Trait Anxiety Inventory (STAI) [38], with a threshold of 39–40 for identifying clinically significant anxiety symptoms [39].

### Statistical analysis

Statistical analyses were conducted in R, version 4.2.1. results [40]. Results were considered significant at α =0.05. We compared baseline characteristics between treatment groups using Pearson's *χ*^2^ tests and Wilcoxon-rank-sum tests as appropriate. To reduce the effect of extreme test performances, we identified values below the 1% and above the 99% percentile on each task and set them to the respective percentile values (winsorization).

To assess potential treatment effects of active tDCS on cognitive performance, we fitted linear mixed models using the lme4 package [41] to predict change from baseline to week 6 on each cognitive test. Treatment group (active tDCS versus sham tDCS) was included as a fixed effect while controlling for the respective baseline cognitive test score (formula: change in cognitive performance ~ treatment group + baseline cognitive performance). Sensitivity analyses included additional models with sex, age, and baseline MADRS as covariates.

To assess potential predictive influences of baseline cognitive performance on antidepressant treatment effects of active tDCS, we again fitted linear mixed models to predict change from baseline to week 6 on the MADRS. Treatment group, performance on the respective cognitive domain, and their interaction were included as fixed effects while controlling for baseline MADRS scores (formula: MADRS change ~ treatment group x cognitive performance at baseline + baseline MADRS score).

All models included the treatment site as a random effect (formula: ~ 1| site). Significance of the model factors was determined using omnibus tests (Type III ANOVA) with Satterthwaite approximation to degrees of freedom. We did not use imputation since linear mixed models are able to handle missing data. Standardized effect sizes for regression coefficients were computed using the emmeans::eff_size() approach, with the sigma parameter being directly extracted from the regression model [42]. We corrected for multiple testing across predictors using the false-discovery-rate (FDR) method [43].

## Results

### Sample characteristics

We analyzed data from 101 patients (active tDCS, n = 50; sham tDCS, n = 51). Mean age (active tDCS 39 [SD 14]; sham tDCS 39 [SD 14]; p = 0.76). Sex: active tDCS 40% male; sham tDCS 40% male. Primary baseline and clinical features across the active and sham-tDCS groups were similar (Table 1 and Supplementary Table 1). Winsorized mean test performances and the number of winsorized measurements per cognitive test are reported in supplementary Table 5 and 6.

**Table 1:** Baseline patient characteristics

Characteristic	tDCS, *n* = 50^1^	Sham, *n* = 51^1^	*p* value^2^
Sex			0.76
Female	30 (60%)	29 (57%)	
Male	20 (40%)	22 (43%)	
Age (years)	39 (14)	39 (14)	0.98
Age of onset of depression (years)	32 (12)	34 (15)	0.85
Duration of current episode (weeks)	62 (69)	58 (69)	0.66
Schooling (years)	11.84 (1.93)	11.66 (1.72)	0.56
MADRS score	22.8 (6.1)	23.2 (5.3)	0.60
BDI score	27 (12)	28 (11)	0.52
WHO/DAS score	22 (9)	24 (11)	0.32
GAF score	55 (10)	56 (9)	0.98
SHAPS-D score	4.6 (3.0)	5.7 (3.5)	0.14
State-trait anxiety inventory state score	53 (11)	55 (9)	0.53
State-trait anxiety inventory trait score	57 (10)	55 (10)	0.73
CD-RISC score	16 (7)	17 (7)	0.68

^1^ *n* (%); mean (SD). ^2^ Pearson’s Chi-squared test; Wilcoxon rank sum test

*MADRS *Montgomery–Åsberg Depression Rating Scale, *BID *Beck Depression Inventory, *WHO/DAS *The World Health Organization Disability Assessment Schedule, *GAF *Global Assessment of Functioning, *SHAPS-D *self- reported anhedonia assessed with the Snaith Hamilton Anhedonia Pleasure Scale, *CD-RISC *Connor-Davidson Resilience Scale

### Treatment effects on neurocognitive test scores

**Fig. 1:**
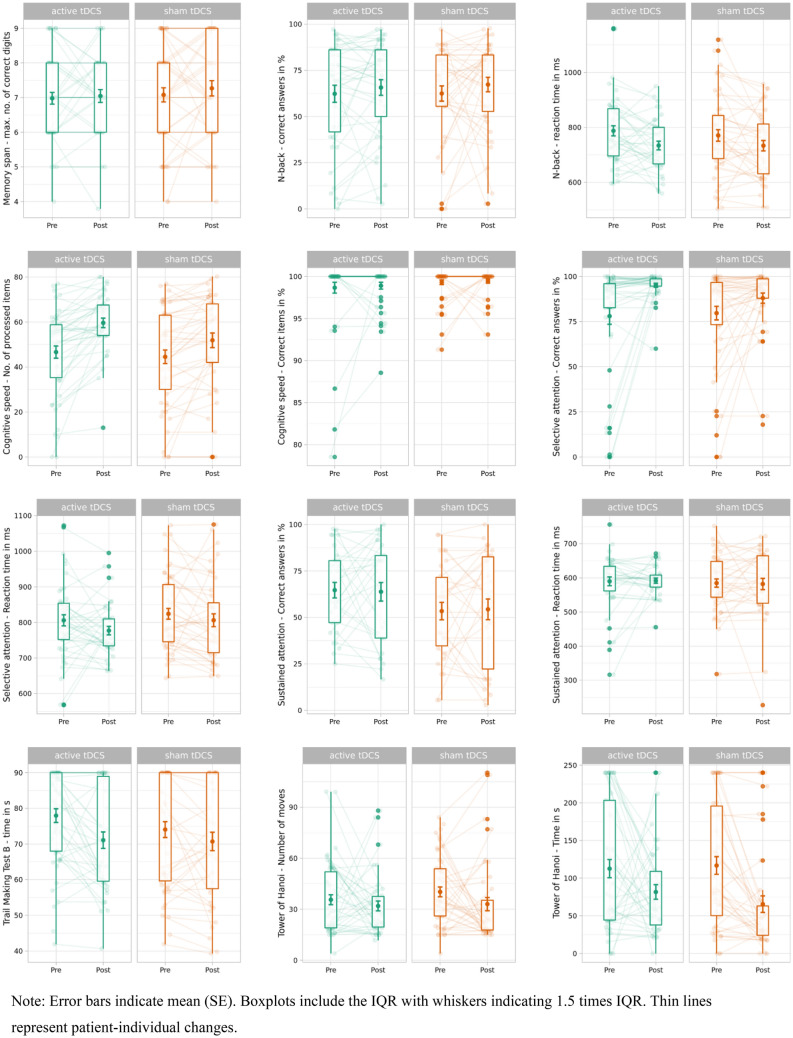
Pre- and post-treatment performance across neurocognitive tests for active tDCS and sham tDCS. Note: Error bars indicate mean (SE). Boxplots include the IQR with whiskers indicating 1.5 times IQR. Thin lines represent patient-individual changes

We observed no significant group-by-time interactions between treatment group and memory span, working memory, selective attention, sustained attention, executive function, or processing speed. Pre- and post-treatment performance across neurocognitive tests for active tDCS and sham tDCS is shown in Fig. 1, and Table 2 provides further statistical information. Results for additional models including sex, age and baseline MADRS yielded similar results (supplementary Table 2–4).

**Table 2:** Treatment effects on neurocognitive test scores

Cognitive measure	Slope active tDCS (95% CI)	Slope sham tDCS (95% CI)	F (df)	*p*	p_fdr_	Standardized effect size (95% CI)
Memory span (maximum number of correct digits)	0.02 (− 0.63 0.66)	0.20 (− 0.44 0.84)	0.72 (1, 73)	0.40	0.80	− 0.19 (− 0.75, 0.37)
Working memory (correct answers in %)	4.86 (− 9.41, 19.1)	6.68 (− 7.47, 20.8)	0.13 (1, 71)	0.72	0.81	− 0.08 (− 0.66, 0.50)
Working memory (reaction time in ms)	-32.8 (− 109, 43.6)	− 38.4 (− 117 40.2)	0.07 (1, 69)	0.79	0.81	0.06 (− 0.71, 0.83)
Cognitive speed (number of processed items)	10.91 (4.12, 17.7)	5.71 (− 1.19, 12.6)	4.69 (1, 73)	**0.03**	0.18	0.49 (− 0.12, 1.09)
Cognitive speed (correct items in %)	0.22 (− 2.90, 3.34)	0.68 (− 3.31, 4.66)	1 (1, 73)	0.32	0.77	− 0.23 (− 1.86, 1.4)
Selective attention (correct items in %)	14.58 (− 3.33, 32.5)	7.83 (− 14.52, 30.2)	4.88 (1, 77)	**0.03**	0.18	0.50 (− 0.92, 1.91)
Selective attention (reaction time in ms)	− 16.9 (− 126, 92.7)	− 11.5 (− 194, 170.9)	0.08 (1, 73)	0.77	0.81	− 0.07 (− 1.96, 1.83)
Sustained attention (correct items in %)	1.52 (− 19.2, 22.2)	− 1.34 (− 27.5, 24.8)	0.18 (1, 56)	0.67	0.81	0.11 (− 0.87, 1.09)
Sustained attention (reaction time in ms)	16.01 (− 75.4, 107)	2.85 (− 155.2, 161)	0.47 (1, 61)	0.50	0.81	0.172 (− 1.61, 1.95)
Trail making B (time in s)	− 5.69 (− 24.6 13.2	− 2.54 (− 25.1, 20.0)	1.78 (1, 75)	0.19	0.76	− 0.31 (− 2.12, 1.51)
Tower of Hanoi (number of moves)	− 4.2 (− 19.7, 11.3)	− 3.05 (− 19.6, 13.5)	0.06 (1, 74)	0.81	0.81	− 0.05 (− 0.73, 0.63)
Tower of Hanoi (time in s)	− 29.8 (− 112, 52.9)	− 44.0 (− 158, 70.4)	1.02 (1, 74)	0.32	0.77	0.23 (− 1.31, 1.77)

*p* values computed using Type III analyses of variance with Satterthwaite's method. Slope active tDCS = standardized slope parameter for active tDCS. Slope sham tDCS = standardized slope parameter for sham tDCS

### Prediction of clinician-rated depression (MADRS)

**Fig. 2:**
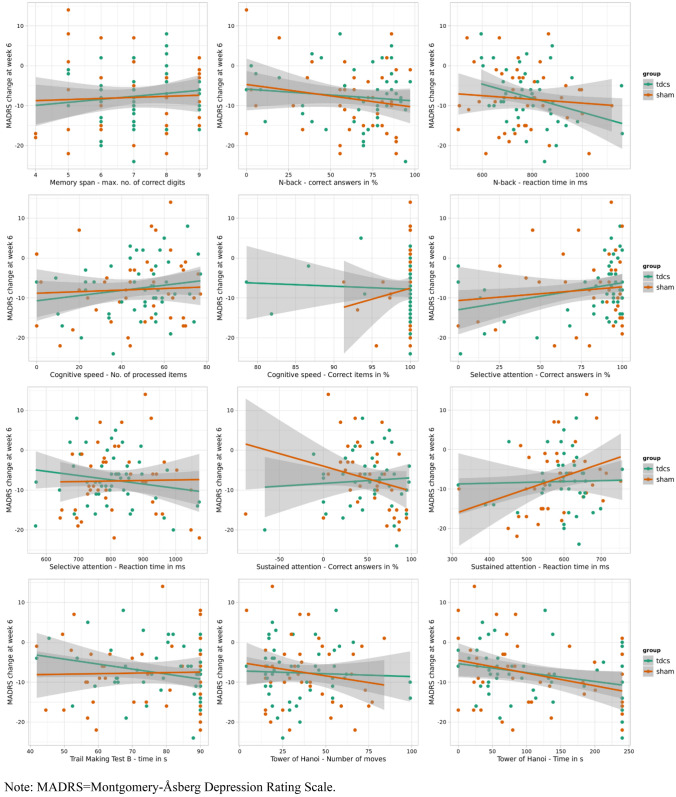
Association between baseline cognitive performance and MADRS change across the trial. *MADRS* Montgomery-Åsberg Depression Rating Scale

We did not detect significant interactions, when predicting MADRS change, between treatment group and memory span, working memory, selective attention, sustained attention, executive function, or processing speed. Table 3 provides the effect size of each neurocognitive test at baseline and Fig. 2 depicts the association between baseline cognitive performance and changes in MADRS scores.

**Table 3:** Prediction of changes MADRS

Measure	Cognitive tests							
	Group		Cognitive test score		Group × cognitive test score			
	F (df)	*p*	F (df)	*p*	F (df)	*p*	p_FDR_	η^2^
Memory span (maximum number of correct digits)	0.12 (1, 89)	0.74	0.66 (1, 89)	0.42	0.14 (1, 89)	0.71	0.71	0.001
Working memory (correct answers in %)	0.20 (1, 88)	0.65	2.66 (1, 88)	0.11	0.42 (1, 88)	0.52	0.63	0.005
Working memory (reaction time in ms)	1.54 (1, 81)	0.22	3.10 (1, 64)	0.08	1.43 (1, 81)	0.24	0.63	0.02
Cognitive speed (number of processed items)	0.29 (1, 89)	0.59	0.87 (1, 88)	0.35	0.43 (1, 89)	0.51	0.63	0.005
Cognitive speed (correct items in %)	0.93 (1, 85)	0.34	0,87 (1, 85)	0.35	0.92 (1, 85)	0.34	0.63	0.01
Selective attention (correct items in %)	0.32 (1, 89)	0.57	3.77 (1, 88)	0.06	0.47 (1, 89)	0.49	0.63	0.005
Selective attention (reaction time in ms)	0.40 (1, 85)	0.53	0.42 (1, 85)	0.52	0.40 (1, 84)	0.53	063	0.005
Sustained attention (correct items in %)	0.66 (1,73)	0.42	0.03 (1, 75)	0.86	0.62 (1, 74)	0.43	0.63	0.008
Sustained attention (reaction time in ms)	1.17 (1, 73)	0.28	2.24 (1, 74)	0.14	1.36 (1, 73)	0.25	0.63	0.02
Trail Making B (time in s)	1.59 (1, 85)	0.21	0.80 (1, 87)	0.37	1.59 (1, 85)	0.21	0.63	0.02
Tower of Hanoi (number of moves)	0.47 (1, 88)	0.49	1.37 (1, 88)	0.25	0.55 (1, 88)	0.46	0.63	0.006
Tower of Hanoi (time in s)	0.12 (1, 87)	0.73	8.32 (1, 87)	0.005	0.031 (1, 87)	0.58	0.63	0.004

*p* values computed using Type III analyses of variance with Satterthwaite's method. MADRS = Montgomery-Åsberg Depression Rating Scale. η^2^ = 0.01 ≤ 0.06 (small effect), 0.06 ≤ 0.14 (moderate effect) and ≥ 0.14 (large effect)

## Discussion

In this ancillary analysis of the DepressionDC trial, a randomized, sham-controlled multicenter study assessing the antidepressant efficacy of a prefrontal tDCS as acute treatment in patients with MDD and SSRI treatment, we found no statistically significant group differences between active tDCS and sham tDCS for the change of performance in FPN-associated cognitive domains (i.e. memory span, working memory, selective attention, sustained attention, executive function and processing speed) from baseline to week 6. Furthermore, baseline performance in these domains was not differentially associated with a change in depression severity for active tDCS compared to sham tDCS.

Our results are in contrast to a recent meta-analysis that found significant effects of tDCS on working memory and attention [28]. This meta-analysis was based on studies with sample sizes between n = 18 [15] and n = 127 [26] the number of treatment sessions (one [24] up to 22 [26] and tDCS dosages(0.5 mA [21, 27], 1 mA [15, 20] and 2 mA [16–18, 22–26]) was highly heterogeneous. Among single studies included in this meta-analysis, several authors reported an improvement of attention/vigilance, working memory, executive functioning, processing speed, and social cognition [15, 17], spatial working memory [18] or processing speed [16]. However, other studies in this meta-analysis are rather in line with our findings and did not show significant effects of tDCS on performance in neurocognitive domains [20–27]. The ELECT-TDCS trial, a clinical study with identical stimulation parameters and a larger sample size, did not find significant effects on cognition either [26].

There are several potential reasons for these negative findings. First, our multicenter trial tested only one set of tDCS parameters with the aim of reducing depressive symptoms. However, dose–response curves for single domains of neurocognitive performance have not been established. They may be non-linear and could theoretically vary from one domain to another [44, 45] as well as from dose–response curves of antidepressant effects. While being in line with previous studies on antidepressant tDCS, the administered dosage in our trial might have been insufficient to optimally modulate specific prefrontal cognitive functions. Second, the main trial did not show beneficial antidepressant effects of active tDCS over sham tDCS. Thus, the applied tDCS protocol might have also been not potent enough to modulate neuroplasticity changes in general. Third, high levels of arousal, estimated by using the State-Trait Anxiety Inventory (STAI), have been reported to diminish cognitive practice effects elicited by tDCS, [46] underlining the potential role of arousal in shaping responses to neuromodulation. In our study, both groups had high baseline STAI scores, and such high baseline anxiety could have reduced the effects of tDCS on neurocognitive performance. Lastly, several studies have reported that tDCS might only elicit procognitive effects when simultaneously combined with specific cognitive tasks [47–52]. Thus, passive stimulation, as administered in our trial, might not be sufficient to enhance cognition in patients with MDD.

To the best of our knowledge, this is the first study that investigates whether cognition at baseline may be used to predict improvement of depression during a course of tDCS. Our study has multiple strengths. The study followed the highest possible trial design standards by being triple-blinded, placebo-controlled, and multicenter. We applied a tDCS protocol (2 mA, 30 min) established in previous studies which showed a superior antidepressant efficacy of active over sham tDCS, i.e. the SELECT-TDCS [53] and ELECT-TDCS [26] trials, and our data-set is one of the biggest samples in the field to date (n = 101). Furthermore, we used a validated digital assessment battery that has successfully been used in other previous studies [31, 32, 54]. While efforts are being made to digitize previously validated cognitive tests [55, 56], such tools which also reduce documentation errors [57, 58], are still underused.

### Limitations

First, there is no uniform consensus on what neurocognitive tests are better used to evaluate the performance in domains associated with FPN function. Our battery included some of the most common tests and slight variations of them. However, other standardized tests could have a higher sensitivity and specificity for detecting neuromodulation effects on cognitive performance [59]. Second, digital tools present a few caveats such as failure of the equipment, corruption of data, and loss of information when retrieving the data. This limited the availability of data in our study. Third, the evaluation of procognitive effects of tDCS and the potential predictive effects of baseline cognition on treatment response were ancillary investigations. Though this data was well balanced across both conditions, there may be latent selection biases making the sample not representative for the whole study population. In addition, the current analysis was likely underpowered to detect small treatment and prediction effects. Lastly, all patients were on a stable SSRI medication for at least 4 weeks prior to inclusion, but not antidepressant-free. Thus, our conclusions regarding the differential effects of SSRI medication and tDCS on performance in distinct neurocognitive domains are limited.

## Conclusion

In conclusion, our analysis does not support the notion that acute treatment with active tDCS compared to sham tDCS leads to an improvement in FPN-related neurocognitive functions. In addition, neurocognitive functioning at baseline did not predict the change of MADRS scores over the course of tDCS. Future research should aim at identifying tDCS protocols with optimal dose–response curves for effects on specific neurocognitive domains. Most promising candidates could then be further optimized by adjusting parameters at an individual patient's level.

## Supplementary Information

Below is the link to the electronic supplementary material.

Supplementary file1 (DOCX 29 KB)

## Data Availability

The de-identified individual patient data in this paper will be made accessible after its publication for non-commercial academic projects that have a legitimate research topic and a clearly stated hypothesis. If the application is accepted, researchers will be asked to get the study approved by their institution's ethics board. The authors will subsequently provide the de-identified data sets via a safe data transfer system. You may find the DepressionDC research protocol as well as further extra information at https://osf.io/cpw6f/.

## References

[1] Woods AJ et al (2016) A technical guide to tDCS, and related non-invasive brain stimulation tools. Clin Neurophysiol Off J Int Fed Clin Neurophysiol 127(2):1031–1048. 10.1016/j.clinph.2015.11.012 10.1016/j.clinph.2015.11.012 PMC4747791 26652115

[2] Lefaucheur J-P et al (2017) Evidence-based guidelines on the therapeutic use of transcranial direct current stimulation (tDCS). Clin Neurophysiol 128(1):56–92. 10.1016/j.clinph.2016.10.087 27866120 10.1016/j.clinph.2016.10.087

[3] Kaiser RH, Andrews-Hanna JR, Wager TD, Pizzagalli DA (2015) Large-Scale Network Dysfunction in Major Depressive Disorder: A Meta-analysis of Resting-State Functional Connectivity. JAMA Psychiat 72(6):603–611. 10.1001/jamapsychiatry.2015.0071 10.1001/jamapsychiatry.2015.0071 PMC4456260 25785575

[4] Fischer M, Moscovitch M, Alain C (2021) A systematic review and meta-analysis of memory-guided attention: Frontal and parietal activation suggests involvement of fronto-parietal networks. Wiley Interdiscip Rev Cogn Sci. 10.1002/wcs.1546 33099860 10.1002/wcs.1546

[5] Lugtmeijer S, Lammers NA, de Haan EHF, de Leeuw F-E, Kessels RPC (2021) Post-Stroke Working Memory Dysfunction: A Meta-Analysis and Systematic Review. Neuropsychol Rev 31(1):202–219. 10.1007/s11065-020-09462-4 33230717 10.1007/s11065-020-09462-4 PMC7889582

[6] Botdorf M, Riggins T (2018) When less is more: Thinner fronto-parietal cortices are associated with better forward digit span performance during early childhood. Neuropsychologia 121:11–18. 10.1016/j.neuropsychologia.2018.10.020 30393004 10.1016/j.neuropsychologia.2018.10.020 PMC6289754

[7] Sauseng P, Klimesch W, Schabus M, Doppelmayr M (2005) Fronto-parietal EEG coherence in theta and upper alpha reflect central executive functions of working memory. Int J Psychophysiol Off J Int Organ Psychophysiol 57(2):97–103. 10.1016/j.ijpsycho.2005.03.018 10.1016/j.ijpsycho.2005.03.018 15967528

[8] Imms P et al (2021) Navigating the link between processing speed and network communication in the human brain. Brain Struct Funct 226(4):1281–1302. 10.1007/s00429-021-02241-8 33704578 10.1007/s00429-021-02241-8

[9] Swick D, Ashley V, Turken U (2011) Are the neural correlates of stopping and not going identical? Quantitative meta-analysis of two response inhibition tasks. Neuroimage 56(3):1655–1665. 10.1016/j.neuroimage.2011.02.070 21376819 10.1016/j.neuroimage.2011.02.070

[10] Rutherford BR et al (2021) Slowed Processing Speed Disrupts Patient Expectancy in Late Life Depression. Am J Geriatr Psychiatry Off J Am Assoc Geriatr Psychiatry. 10.1016/j.jagp.2020.11.001 10.1016/j.jagp.2020.11.001 PMC8099936 33250338

[11] Gass CS, Patten B (2020) Depressive symptoms, memory complaints, and memory test performance. J Clin Exp Neuropsychol 42(6):602–610. 10.1080/13803395.2020.1782848 32752927 10.1080/13803395.2020.1782848

[12] Dotson VM et al (2020) Depression and Cognitive Control across the Lifespan: a Systematic Review and Meta-Analysis. Neuropsychol Rev 30(4):461–476. 10.1007/s11065-020-09436-6 32385756 10.1007/s11065-020-09436-6 PMC9637269

[13] Nikolin S, Tan YY, Schwaab A, Moffa A, Loo CK, Martin D (2021) An investigation of working memory deficits in depression using the n-back task: A systematic review and meta-analysis. J Affect Disord 284:1–8. 10.1016/j.jad.2021.01.084 33581489 10.1016/j.jad.2021.01.084

[14] Woolridge SM, Harrison GW, Best MW, Bowie CR (2021) Attention bias modification in depression: A randomized trial using a novel, reward-based, eye-tracking approach. J Behav Ther Exp Psychiatry. 10.1016/j.jbtep.2020.101621 33202263 10.1016/j.jbtep.2020.101621

[15] Fregni F, Boggio PS, Nitsche MA, Rigonatti SP, Pascual-Leone A (2006) Cognitive effects of repeated sessions of transcranial direct current stimulation in patients with depression. Depress Anxiety 23(8):482–484. 10.1002/da.20201 16845648 10.1002/da.20201

[16] Loo CK, Alonzo A, Martin D, Mitchell PB, Galvez V, Sachdev P (2012) Transcranial direct current stimulation for depression: 3-week, randomised, sham-controlled trial. Br J Psychiatry 200(1):52–59. 10.1192/bjp.bp.111.097634 22215866 10.1192/bjp.bp.111.097634

[17] Boggio PS et al (2007) Go-no-go task performance improvement after anodal transcranial DC stimulation of the left dorsolateral prefrontal cortex in major depression. J Affect Disord 101(1–3):91–98. 10.1016/j.jad.2006.10.026 17166593 10.1016/j.jad.2006.10.026

[18] Salehinejad MA, Ghanavai E, Rostami R, Nejati V (2017) Cognitive control dysfunction in emotion dysregulation and psychopathology of major depression (MD): Evidence from transcranial brain stimulation of the dorsolateral prefrontal cortex (DLPFC). J Affect Disord 210:241–248. 10.1016/j.jad.2016.12.036 28064113 10.1016/j.jad.2016.12.036

[19] Boggio PS et al (2008) A randomized, double-blind clinical trial on the efficacy of cortical direct current stimulation for the treatment of major depression. Int J Neuropsychopharmacol 11(2):249–254. 10.1017/S1461145707007833 17559710 10.1017/S1461145707007833 PMC3372849

[20] Loo CK et al (2010) A double-blind, sham-controlled trial of transcranial direct current stimulation for the treatment of depression. Int J Neuropsychopharmacol 13(01):61. 10.1017/S1461145709990411 19671217 10.1017/S1461145709990411

[21] Palm U et al (2012) Transcranial direct current stimulation in treatment resistant depression: A randomized double-blind, placebo-controlled study. Brain Stimulat 5(3):242–251. 10.1016/j.brs.2011.08.005 10.1016/j.brs.2011.08.005 21962978

[22] Segrave RA, Arnold S, Hoy K, Fitzgerald PB (2014) Concurrent Cognitive Control Training Augments the Antidepressant Efficacy of tDCS: A Pilot Study. Brain Stimulat 7(2):325–331. 10.1016/j.brs.2013.12.008 10.1016/j.brs.2013.12.008 24486425

[23] Bennabi D et al (2015) Pilot study of feasibility of the effect of treatment with tDCS in patients suffering from treatment-resistant depression treated with escitalopram. Clin Neurophysiol Off J Int Fed Clin Neurophysiol 126(6):1185–1189. 10.1016/j.clinph.2014.09.026 10.1016/j.clinph.2014.09.026 25454337

[24] Moreno ML et al (2015) Effects of acute transcranial direct current stimulation in hot and cold working memory tasks in healthy and depressed subjects. Neurosci Lett 591:126–131. 10.1016/j.neulet.2015.02.036 25708347 10.1016/j.neulet.2015.02.036

[25] Brunoni AR, Tortella G, Benseñor IM, Lotufo PA, Carvalho AF, Fregni F (2016) Cognitive effects of transcranial direct current stimulation in depression: Results from the SELECT-TDCS trial and insights for further clinical trials. J Affect Disord 202:46–52. 10.1016/j.jad.2016.03.066 27253216 10.1016/j.jad.2016.03.066

[26] Brunoni AR et al (2017) Trial of Electrical Direct-Current Therapy versus Escitalopram for Depression. N Engl J Med 376(26):2523–2533. 10.1056/NEJMoa1612999 28657871 10.1056/NEJMoa1612999

[27] Pavlova EL et al (2018) Transcranial direct current stimulation of 20- and 30-minutes combined with sertraline for the treatment of depression. Prog Neuropsychopharmacol Biol Psychiatry 82:31–38. 10.1016/j.pnpbp.2017.12.004 29233783 10.1016/j.pnpbp.2017.12.004

[28] Begemann MJ, Brand BA, Ćurčić-Blake B, Aleman A, Sommer IE (2020) Efficacy of non-invasive brain stimulation on cognitive functioning in brain disorders: a meta-analysis. Psychol Med 50(15):2465–2486. 10.1017/S0033291720003670 33070785 10.1017/S0033291720003670 PMC7737055

[29] Burkhardt G et al (2023) Transcranial direct current stimulation as an additional treatment to selective serotonin reuptake inhibitors in adults with major depressive disorder in Germany (DepressionDC): a triple-blind, randomised, sham-controlled, multicentre trial. Lancet Lond Engl. 10.1016/S0140-6736(23)00640-2 10.1016/S0140-6736(23)00640-2 37414064

[30] Fuge P et al (2014) Assessment of Age-related Changes in Cognitive Functions Using EmoCogMeter, a Novel Tablet-computer Based Approach. J Vis Exp JoVE 84:50942. 10.3791/50942 10.3791/50942 PMC4123685 24561917

[31] Fuge P et al (2014) Interaction of Early Life Stress and Corticotropin-Releasing Hormone Receptor Gene: Effects on Working Memory. Biol Psychiatry 76(11):888–894. 10.1016/j.biopsych.2014.04.016 24931706 10.1016/j.biopsych.2014.04.016

[32] Grimm S et al (2015) Variation in the corticotropin-releasing hormone receptor 1 (CRHR1) gene modulates age effects on working memory. J Psychiatr Res 61:57–63. 10.1016/j.jpsychires.2014.12.001 25541005 10.1016/j.jpsychires.2014.12.001

[33] Schroeder RW, Twumasi-Ankrah P, Baade LE, Marshall PS (2012) Reliable Digit Span: A Systematic Review and Cross-Validation Study. Assessment 19(1):21–30. 10.1177/1073191111428764 22156721 10.1177/1073191111428764

[34] Epp AM, Dobson KS, Dozois DJA, Frewen PA (2012) A systematic meta-analysis of the Stroop task in depression. Clin Psychol Rev 32(4):316–328. 10.1016/j.cpr.2012.02.005 22459792 10.1016/j.cpr.2012.02.005

[35] Bowie CR, Harvey PD (2006) Administration and interpretation of the Trail Making Test. Nat Protoc 1(5):2277–2281. 10.1038/nprot.2006.390 17406468 10.1038/nprot.2006.390

[36] Simon HA (1975) The functional equivalence of problem solving skills. Cognit Psychol 7(2):268–288. 10.1016/0010-0285(75)90012-2

[37] Montgomery SA, Asberg M (1979) A new depression scale designed to be sensitive to change. Br J Psychiatry J Ment Sci 134:382–389. 10.1192/bjp.134.4.382 10.1192/bjp.134.4.382 444788

[38] Julian LJ (2011) Measures of anxiety: State-Trait Anxiety Inventory (STAI), Beck Anxiety Inventory (BAI), and Hospital Anxiety and Depression Scale-Anxiety (HADS-A). Arthritis Care Res. 10.1002/acr.20561 10.1002/acr.20561 PMC3879951 22588767

[39] Knight RG, Waal-Manning HJ, Spears GF (1983) Some norms and reliability data for the State-Trait Anxiety Inventory and the Zung Self-Rating Depression scale. Br J Clin Psychol 22(Pt 4):245–249. 10.1111/j.2044-8260.1983.tb00610.x 6640176 10.1111/j.2044-8260.1983.tb00610.x

[40] “R: a language and environment for statistical computing.” Accessed: Jul. 10, 2023. [Online]. Available: https://www.gbif.org/tool/81287/r-a-language-and-environment-for-statistical-computing

[41] Bates D, Mächler M, Bolker B, Walker S (2015) Fitting Linear Mixed-Effects Models Using lme4. J Stat Softw 67:1–48. 10.18637/jss.v067.i01

[42] R Lenth (2016) Least-Squares Means: The R Package lsmeans, J Stat Softw 10.18637/jss.v069.i01

[43] Benjamini Y, Hochberg Y (1995) Controlling the False Discovery Rate: A Practical and Powerful Approach to Multiple Testing. J R Stat Soc Ser B Methodol 57(1):289–300. 10.1111/j.2517-6161.1995.tb02031.x

[44] Ehrhardt SE, Filmer HL, Wards Y, Mattingley JB, Dux PE (2021) The influence of tDCS intensity on decision-making training and transfer outcomes. J Neurophysiol 125(2):385–397. 10.1152/jn.00423.2020 33174483 10.1152/jn.00423.2020

[45] Ehrhardt SE, Ballard T, Wards Y, Dux PE, Filmer HL (2022) tDCS augments decision-making efficiency in an intensity dependent manner: A training study. Neuropsychologia 176:108397. 10.1016/j.neuropsychologia.2022.108397 36272676 10.1016/j.neuropsychologia.2022.108397

[46] Esposito M, Ferrari C, Fracassi C, Miniussi C, Brignani D (2022) Responsiveness to left-prefrontal tDCS varies according to arousal levels. Eur J Neurosci 55(3):762–777. 10.1111/ejn.15584 34978110 10.1111/ejn.15584 PMC9302688

[47] Schneider N et al (2021) Combining transcranial direct current stimulation with a motor-cognitive task: the impact on dual-task walking costs in older adults. J Neuroengineering Rehabil 18(1):23. 10.1186/s12984-021-00826-2 10.1186/s12984-021-00826-2 PMC7852224 33526043

[48] Lo KYH et al (2022) Concurrent anodal transcranial direct current stimulation (tDCS) with cognitive training to improve cognition in schizophrenia. Schizophr Res 241:184–186. 10.1016/j.schres.2022.01.026 35131597 10.1016/j.schres.2022.01.026

[49] Filmer HL, Lyons M, Mattingley JB, Dux PE (2017) Anodal tDCS applied during multitasking training leads to transferable performance gains. Sci Rep 7(1):12988. 10.1038/s41598-017-13075-y 29021526 10.1038/s41598-017-13075-y PMC5636876

[50] Oldrati V, Colombo B, Antonietti A (2018) Combination of a short cognitive training and tDCS to enhance visuospatial skills: A comparison between online and offline neuromodulation. Brain Res 1678:32–39. 10.1016/j.brainres.2017.10.002 29017911 10.1016/j.brainres.2017.10.002

[51] Han YMY et al (2022) Neurophysiological and behavioral effects of multisession prefrontal tDCS and concurrent cognitive remediation training in patients with autism spectrum disorder (ASD): A double-blind, randomized controlled fNIRS study. Brain Stimulat 15(2):414–425. 10.1016/j.brs.2022.02.004 10.1016/j.brs.2022.02.004 35181532

[52] Weller S, Nitsche MA, Plewnia C (2020) Enhancing cognitive control training with transcranial direct current stimulation: a systematic parameter study. Brain Stimulat 13(5):1358–1369. 10.1016/j.brs.2020.07.006 10.1016/j.brs.2020.07.006 32687899

[53] Brunoni AR et al (2013) The Sertraline vs Electrical Current Therapy for Treating Depression Clinical Study: Results From a Factorial, Randomized, Controlled Trial. JAMA Psychiat 70(4):383–391. 10.1001/2013.jamapsychiatry.32 10.1001/2013.jamapsychiatry.32 23389323

[54] Aust S et al (2022) Efficacy of Augmentation of Cognitive Behavioral Therapy With Transcranial Direct Current Stimulation for Depression: A Randomized Clinical Trial. JAMA Psychiat 79(6):528–537. 10.1001/jamapsychiatry.2022.0696 10.1001/jamapsychiatry.2022.0696 PMC9021985 35442431

[55] Vermeent S, Dotsch R, Schmand B, Klaming L, Miller JB, van Elswijk G (2020) Evidence of Validity for a Newly Developed Digital Cognitive Test Battery. Front Psychol 11:770. 10.3389/fpsyg.2020.00770 32390918 10.3389/fpsyg.2020.00770 PMC7194127

[56] Chan JYC et al (2022) Evaluation of Digital Drawing Tests and Paper-and-Pencil Drawing Tests for the Screening of Mild Cognitive Impairment and Dementia: A Systematic Review and Meta-analysis of Diagnostic Studies. Neuropsychol Rev 32(3):566–576. 10.1007/s11065-021-09523-2 34657249 10.1007/s11065-021-09523-2 PMC9381608

[57] Håkansson I, Lundström M, Stenevi U, Ehinger B (2001) Data reliability and structure in the Swedish National Cataract Register. Acta Ophthalmol Scand 79(5):518–523. 10.1034/j.1600-0420.2001.790519.x 11594992 10.1034/j.1600-0420.2001.790519.x

[58] Point S, Baruch Y (2023) (Re)thinking transcription strategies: Current challenges and future research directions. Scand J Manag 39(2):101272. 10.1016/j.scaman.2023.101272

[59] Parkinson WL, Rehman Y, Rathbone M, Upadhye S (2020) Performances on individual neurocognitive tests by people experiencing a current major depression episode: A systematic review and meta-analysis. J Affect Disord 276:249–259. 10.1016/j.jad.2020.07.036 32697706 10.1016/j.jad.2020.07.036

