# Supplementary material for: Neurocognitive function as outcome and predictor for prefrontal transcranial direct current stimulation in major depressive disorder: an analysis from the DepressionDC trial
Source: Eur Arch Psychiatry Clin Neurosci. 2024 Feb 26;275(6):1715–24. doi: 10.1007/s00406-024-01759-2 (PMC12500756; doi:10.1007/s00406-024-01759-2)
Supplement: Supplementary file 1 — Supplementary file1 (DOCX 29 KB) [file 406_2024_1759_MOESM1_ESM.docx]

**Supplementary Material**

**Inclusion and exclusion criteria**

(1) Inclusion Criteria:

- 18 to 65-year-old men and women.
- Primary DSM-5 diagnosis of Major Depression based on the Structured Clinical Interview for DSM-5 Axis I Disorders, Research Version (SCID-5-RV) with a single or recurrent episode with a duration of 4 weeks.
- Current depressed episode has a duration of less than five years.
- Baseline Hamilton Depression Rating Scale (HDRS) score ≥15
- In the present episode, the patient did not respond to at least one antidepressant therapy, which is defined as a minimum level of 2 on the Antidepressant Treatment History Form (ATHF) and a maximum of four antidepressant medication trials of a sufficient dosage and duration.
- At the present episode, the patient is taking an SSRI at a sufficient dosage for at least four weeks (defined as a minimum level of 2 on the ATHF).
- Patient is capable and willing to provide informed consent.
- The patient has a negative pregnancy test and is willing to use contraception during study treatment for women with reproductive potential (i.e., less than 2 years postmenopausal).

(2) Exclusion criteria:

- Researchers, site staff members who are specifically involved in this project, and members of their immediate families (defined as a spouse, parent, child, or sibling, whether by natural birth or legal adoption).
- Acute risk for suicide as indicated by a score of >4 on item 10 of the MADRS or by agreement with items 4 and/or 5 of the C-SSRS.
- A high degree of therapy resistance, indicated by more than four adequate treatment efforts made during the current episode, each with an ATHF score of at least 3.
- In the current depressive episode, having had or being treated with electroconvulsive therapy.
- Previous treatment with deep brain stimulation, vagus nerve stimulation, and/or other intracranial implants (clips, cochlear implants).
- Any further pertinent axis-I and/or axis-II psychiatric disorders as determined by the Mini-International Neuropsychiatric Interview (M.I.N.I.) and the Structured Clinical Interview for DSM-5 II (SCID-II).
- Any relevant non-treated and unstable medical condition as determined by a physician.
- Previous treatment with tDCS (excluding single experimental tDCS sessions).
- Pregnancy.

**Baseline Characteristics**

Supplementary Table 1. Pre- and post-neurocognitive test performance of patients with available Emocogmeter data.

| Characteristic | tDCS, n = 50^1^ | Sham, n = 51^1^ | p-value^2^ |
| --- | --- | --- | --- |
| Baseline memory span (maximum number of correct digits) | 6.98 (1.20) | 7.06 (1.50) | 0.63 |
| Post memory span (maximum number of correct digits) | 7.03 (1.22) | 7.27 (1.41) | 0.42 |
| Baseline working memory (correct answers in %) | 62 (29) | 62 (27) | 0.75 |
| Post working memory (correct answers in %) | 66 (26) | 61 (40) | 0.85 |
| Baseline working memory (reaction time in ms) | 788 (128) | 769 (147) | 0.55 |
| Post working memory (reaction time in ms) | 734 (99) | 734 (123) | 0.95 |
| Baseline cognitive speed (number of processed items) | 47 (19) | 45 (21) | 0.61 |
| Post cognitive speed (number of processed items) | 60 (13) | 52 (21) | 0.15 |
| Baseline cognitive speed (correct items in %) | 97.06 (14.55) | 99.30 (1.88) | 0.64 |
| Post cognitive speed (correct items in %) | 98.51 (4.53) | 99.45 (1.54) | 0.35 |
| Baseline selective attention (correct items in %) | 78 (32) | 80 (27) | 0.85 |
| Post selective attention (correct items in %) | 95 (7) | 88 (20) | **0.02** |
| Baseline selective attention (reaction time in ms) | 805 (109) | 825 (107) | 0.54 |
| Post selective attention (reaction time in ms) | 777 (75) | 807 (118) | 0.37 |
| Baseline sustained attention (correct items in %) | 51 (35) | 46 (49) | 0.75 |
| Post sustained attention (correct items in %) | 57 (30) | 50 (38) | 0.56 |
| Baseline sustained attention (reaction time in ms) | 590 (85) | 585 (82) | 0.39 |
| Post sustained attention (reaction time in ms) | 592 (44) | 576 (127) | 0.86 |
| Baseline Trail Making B (time in s) | 78 (13) | 74 (16) | 0.43 |
| Post Trail Making B (time in s) | 71 (14) | 71 (17) | 1.00 |
| Baseline Tower of Hanoi (number of moves) | 36 (22) | 40 (20) | 0.21 |
| Post Tower of Hanoi (number of moves) | 32 (18) | 0.52 | 0.52 |
| Baseline Tower of Hanoi (time in s) | 113 (84) | 117 (83) | 0.79 |
| Post Tower of Hanoi (time in s) | 82 (61) | 65 (70) | **0.04** |

^1^ n (%); mean (SD). ^2^ Pearson’s Chi-squared test; Wilcoxon rank sum test.

Supplementary Table 2. Treatment effects with sex as a covariate

| Cognitive measure | Slope active tDCS (95% CI) | Slope sham tDCS (95% CI) | F (df) | p | p_fdr_ | Standardized effect size (95% CI) |
| --- | --- | --- | --- | --- | --- | --- |
| Cognitive speed (correct items in %) | 0.15 (-4.33, 4.63) | 0.65 (-4.79, 6.08) | 1.17 (1, 72) | 0.28 | 0.67 | -0.25 (-2.42, 1.92) |
| Cognitive speed (number of processed items) | 11.13 (3.72, 18.5) | 5.88 (-1.64, 13.4) | 4.71 (1, 72) | **0.03** | 0.18 | 0.49 (-0.15, 1.12) |
| Memory span (maximum number of correct digits) | 0.03 (-0.64, 0.70) | 0.21 (-0.45, 0.87) | 0.68 (1, 72) | 0.41 | 0.70 | -0.19 (-0.75, 0.38) |
| Working memory (correct answers in %) | 5.99 (-9.12, 21.1) | 7.76 (-7.14, 22.7) | 0.13 (1, 70) | 0.72 | 0.76 | -0.08 (-0.65, 0.49) |
| Working memory (reaction time in ms) | -29.9 (-153, 92.9) | -36.4 (-160, 87.4) | 0.09 (1, 68) | 0.76 | 0.76 | 0.07 (-1.07, 1.21) |
| Selective attention (correct items in %) | 14.30 (-10.3, 38.9) | 7.69 (-22.5, 37.9) | 4.63 (1, 76) | **0.03** | 0.18 | 0.48 (-1.36, 2.33) |
| Selective attention (reaction time in ms) | -15.4 (-156, 125) | -10.6 (-257, 235) | 0.07 (1, 72) | 0.80 | 0.32 | -0.06 (-2.54, 2.42) |
| Sustained attention (correct items in %) | 2.65 (-21.7, 27.0) | -1.10 (-30.4, 28.2) | 0.30 (1, 55) | 0.58 | 0.76 | 0.15 (-0.94, 1.23) |
| Sustained attention (reaction time in ms) | 12.10 (-135, 159) | 0.81 (-285, 286) | 0.35 (1, 60) | 0.56 | 0.76 | 0.15 (-2.89, 3.19) |
| Tower of Hanoi (number of moves) | -5.82 (-29.7, 18.1) | -4.29 (-31.9, 23.3) | 0.10 (1, 73) | 0.76 | 0.76 | -0.07 (-1.13, 0.99) |
| Tower of Hanoi (time in s) | -31.6 ( -141, 77.4) | -45.1 (-199, 108.5) | 0.92 (1, 73) | 0.34 | 0.68 | 0.22 (-1.79, 2.23) |
| Trail making B (time in s) | -5.64 (-31.4, 20.1) | -2.52 (-32.5, 27.5) | 1.72 (1, 74) | 0.19 | 0.57 | -0.30 (-2.63, 2.03) |

Supplementary Table 3. Treatment effects with age as a covariate

| Cognitive measure | Slope active tDCS (95% CI) | Slope sham tDCS (95% CI) | F (df) | p | p_fdr_ | Standardized effect size (95% CI) |
| --- | --- | --- | --- | --- | --- | --- |
| Cognitive speed (correct items in %) | 0.22 (-3.05, 3.48) | 0.68 (-3.64, 5.00) | 1.10 (1, 72) | 0.30 | 0.72 | -0.24 (-2.02, 1.54) |
| Cognitive speed (number of processed items) | 11.68 (-1.86, 25.2) | 6.22 (-12.42, 24.8) | 5.93 (1, 76) | **0.02** | 0.18 | 0.55 (-1.05, 2.14) |
| Memory span (maximum number of correct digits) | 0.04 (-0.66, 0.74) | 0.23 (-0.46, 0.92) | 0.72 (1, 72) | 0.40 | 0.80 | -0.19 (-0.76, 0.37) |
| Working memory (correct answers in %) | 5.28 (-9.12, 19.7) | 7.34 (-6.95, 21.6) | 0.17 (1, 70) | 0.68 | 0.82 | -0.09 (-0.68, 0.49) |
| Working memory (reaction time in ms) | -54.7 (-134, 25.0) | -60.3 (-139, 18.3) | 0.09 (1, 63) | 0.77 | 0.84 | 0.07 (-0.52, 0.66) |
| Selective attention (correct items in %) | 14.49 (-3.9, 32.9) | 7.92 (-16.3, 32.1) | 4.89 (1, 76) | **0.03** | 0.18 | 0.50 (-1.06, 2.05) |
| Selective attention (reaction time in ms) | -18.50 (-131, 94.5) | -9.96 (-189, 169.5) | 0.23 (1, 72) | 0.63 | 0.82 | -0.11 (-2.07, 1.84) |
| Sustained attention (correct items in %) | 1.49 (-21.4, 24.4) | -1.31 (-32.3, 29.6) | 0.17 (1, 55) | 0.68 | 0.82 | 0.11 (-1.02, 1.24) |
| Sustained attention (reaction time in ms) | 15.21 (-82.1, 113) | 3.56 (-230.9, 238) | 0.36 (1, 60) | 0.55 | 0.82 | 0.15 (-2.41, 2.72) |
| Tower of Hanoi (number of moves) | -4.61 (-22.4, 13.2) | -3.67 (-23.8, 16.4) | 0.04 (1, 73) | 0.85 | 0.85 | -0.04 (-0.84, 0.75) |
| Tower of Hanoi (time in s) | -29.2 (-117, 58.2) | -44.5 (-180, 91.0) | 1.20 (1, 73) | 0.28 | 0.72 | 0.25 (-1.57, 2.07) |
| Trail making B (time in s) | -5.48 (-23.5, 12.5 | -2.74 (-28.8, 23.3) | 1.32 (1, 74) | 0.25 | 0.72 | -0.27 (-2.33, 1.79) |

Supplementary Table 4. Treatment effects with baseline MADRS as a covariate

| Cognitive measure | Slope active tDCS (95% CI) | Slope sham tDCS (95% CI) | F (df) | p | p_fdr_ | Standardized effect size (95% CI) |
| --- | --- | --- | --- | --- | --- | --- |
| Cognitive speed (correct items in %) | 0.22 (-3.57, 4.01) | 0.68 (-3.87, 5.22) | 0.99 (1, 72) | 0.32 | 0.78 | -0.23 (-2.07, 1.61) |
| Cognitive speed (number of processed items) | 11.04 (3.75, 18.3) | 5.78 (-1.55, 13.1) | 4.70 (1, 72) | **0.03** | 0.18 | 0.49 (-0.16, 1.12) |
| Memory span (maximum number of correct digits) | 0.003 (-0.68, 0.69) | 0.19 (-0.48, 0.86) | 0.73 (1, 72) | 0.39 | 0.78 | -0.19 (-0.76, 0.38) |
| Working memory (correct answers in %) | 3.53 (-11.52, 18.6) | 5.78 (-8.93, 20.5) | 0.21 (1, 70) | 0.65 | 0.85 | -0.10 (-0.69, 0.49) |
| Working memory (reaction time in ms) | -31.4 (-126, 63.0) | -37.9 (-129, 53.2) | 0.10 (1, 68) | 0.76 | 0.85 | 0.07 (-0.83, 0.97) |
| Selective attention (correct items in %) | 14.57 (-7.18, 36.3) | 7.85 (-17.15, 32.8) | 4.77 (1, 76) | **0.03** | 0.18 | 0.49 (-1.08, 2.06) |
| Selective attention (reaction time in ms) | -16.0 (-143, 111) | -12.3 (-197, 172) | 0.04 (1, 72) | 0.85 | 0.85 | -0.05 (-1.97, 1.88) |
| Sustained attention (correct items in %) | 1.75 (-24.9, 28.4) | -1.57 (-30.1, 27.0) | 0.24 (1, 55) | 0.63 | 0.85 | 0.13 (-0.94, 1.2) |
| Sustained attention (reaction time in ms) | 16.15 (-96, 128) | 2.73 (-184, 189) | 0.48 (1, 60) | 0.49 | 0.84 | 0.174 (-1.9, 2.25) |
| Tower of Hanoi (number of moves) | -5.08 (-25.9, 15.8) | -3.73 (-26.6, 19.1) | 0.08 (1, 74) | 0.78 | 0.85 | -0.06 (-0.96, 0.83) |
| Tower of Hanoi (time in s) | -30.3 (-128, 67.4) | -43.5 (-168, 81.3) | 0.89 (1, 73) | 0.35 | 0.78 | 0.22 (-1.47, 1.9) |
| Trail making B (time in s) | -5.68 (-29.1, 17.7) | -2.55 (-28.6, 23.5) | 1.73 (1, 74) | 0.19 | 0.76 | -0.30 (-2.36, 1.76) |

Supplementary Table 5. Winsorized pre- and post-neurocognitive test performance

| Characteristic | tDCS, n = 50^1^ | Sham, n = 51^1^ | p-value^2^ |
| --- | --- | --- | --- |
| Baseline memory span (maximum number of correct digits) | 6.98 (1.20) | 7.08 (1.45) | 0.63 |
| Post memory span (maximum number of correct digits) | 7.03 (1.22) | 7.27 (1.41) | 0.42 |
| Baseline working memory (correct answers in %) | 62 (29) | 62 (27) | 0.75 |
| Post working memory (correct answers in %) | 66 (26) | 61 (40) | 0.85 |
| Baseline working memory (reaction time in ms) | 788 (125) | 771 (144) | 0.55 |
| Post working memory (reaction time in ms) | 734 (99) | 734 (123) | 0.95 |
| Baseline cognitive speed (number of processed items) | 47 (19) | 45 (21) | 0.61 |
| Post cognitive speed (number of processed items) | 60 (13) | 52 (21) | 0.15 |
| Baseline cognitive speed (correct items in %) | 98.67 (4.47) | 99.30 (1.88) | 0.64 |
| Post cognitive speed (correct items in %) | 98.51 (4.53) | 99.45 (1.54) | 0.35 |
| Baseline selective attention (correct items in %) | 78 (32) | 80 (27) | 0.85 |
| Post selective attention (correct items in %) | 95 (7) | 88 (20) | **0.02** |
| Baseline selective attention (reaction time in ms) | 806 (107) | 824 (106) | 0.54 |
| Post selective attention (reaction time in ms) | 777 (75) | 807 (118) | 0.37 |
| Baseline sustained attention (correct items in %) | 51 (35) | 49 (34) | 0.75 |
| Post sustained attention (correct items in %) | 57 (30) | 50 (38) | 0.56 |
| Baseline sustained attention (reaction time in ms) | 590 (83) | 585 (82) | 0.39 |
| Post sustained attention (reaction time in ms) | 592 (44) | 576 (127) | 0.86 |
| Baseline Trail Making B (time in s) | 78 (13) | 74 (16) | 0.43 |
| Post Trail Making B (time in s) | 71 (14) | 71 (17) | 1.00 |
| Baseline Tower of Hanoi (number of moves) | 36 (22) | 40 (20) | 0.21 |
| Post Tower of Hanoi (number of moves) | 32 (18) | 0.52 | 0.52 |
| Baseline Tower of Hanoi (time in s) | 113 (84) | 117 (83) | 0.79 |
| Post Tower of Hanoi (time in s) | 82 (61) | 65 (70) | **0.04** |

^1^ n (%); mean (SD). ^2^ Pearson’s Chi-squared test; Wilcoxon rank sum test.

Supplementary Table 6. Number of winsorized measurements per task, group, and time point.

| Characteristic | tDCS | Sham |
| --- | --- | --- |
| Memory span (maximum number of correct digits) | 0 | 0 |
| Working memory (correct answers in %) | 0 | 1 |
| Working memory (reaction time in ms) | 5 | 4 |
| Cognitive speed (number of processed items) | 1 | 0 |
| Cognitive speed (correct items in %) | 2 | 3 |
| Selective attention (correct items in %) | 0 | 0 |
| Selective attention (reaction time in ms) | 4 | 3 |
| Sustained attention (correct items in %) | 7 | 9 |
| Sustained attention (reaction time in ms) | 11 | 6 |
| Trail Making B (time in s) | 2 | 2 |
| Tower of Hanoi (number of moves) | 2 | 1 |
| Tower of Hanoi (time in s) | 2 | 1 |

**Description of the EmoCogMeter neurocognitive tests**

Memory span: The participants are presented with a series of numerals and subsequently requested to enter them. It begins with two numerals and provides an extended list of numbers if participants are successful. The task concludes after 5 minutes, or earlier if subjects are able to recall nine digits. Recorded are the digit span and the total number of trials required to attain that level. A higher maximum number of correct digits indicates better performance.

Working memory (N-back test): The participants view sequences of positive, negative, and impartial words and respond if a word was presented twice previously. After 5 minutes, the assignment concludes, and the number of correct responses (hits), false responses, and mean response latency are recorded. A reduced time required to complete the task and higher percentage of correct responses indicate better performance.

Selective attention: Subjects are administered a variant of the Stroop task and instructed to respond if the written color name matches the color ink it was displayed in. After 3 minutes, the assignment concludes and the number of correct responses (hits), false responses, missing responses, and mean response latency are recorded. A reduced time required to complete the task and higher percentage of correct responses indicate better performance.

Sustained attention: a task that includes a small working memory component is used. A circle composed of small blue and yellow circles that are illuminated in a pseudo-random configuration is displayed in the center of the screen. The subject is instructed to recognize and respond to blue-yellow-yellow sequences. After four minutes, the assignment concludes, and the number of correct responses (hits), incorrect responses, and omitted responses is recorded. A reduced time required to complete the task and higher percentage of correct responses indicate better performance.

Trail Making B: Subjects are asked to link 13 numerals and 12 characters on the screen in a sequential and alternate manner to test task switching. The test does not show the correct path, nor continues to the next step if the subject makes a mistake. The task ends after 90 seconds if it isn’t completed. A reduced time required to complete the task indicates better performance.

Tower of Hanoi: The ability to plan and accomplish an objective through a series of intermediate actions was evaluated. A version with three smaller rods and four smaller discs was utilized. Subjects were instructed to move the complete collection of discs to another rod in accordance with the following instructions: (1) Only one disk at a time may be relocated. (2) Each motion consists of sliding the upper disk from one rod to another, on top of any other disks that may already be on that rod. (3) A larger disk may not be inserted atop a smaller disk. The test concludes if subjects do not reach the target within four minutes. A reduced number of moves, and time required to complete the task indicate better performance.

Cognitive speed (Symbol letter modality test); In the upper portion of the screen, nine letters and corresponding geometric symbols are assigned to the subject. Subjects are instructed to move the corresponding symbol under each letter as rapidly as possible, while grids of letters are displayed in the lower portion of the screen. The number of correct responses (hits), incorrect responses, missing responses, and mean response latency within the allotted time (90 seconds) are recorded. A higher number of processed items and a reduced time required to complete the task indicate better performance.

**Outcome metrics**

Reaction time = time required to complete an item (successfully or unsuccessfully) within a given task. Measured in milliseconds.

Accuracy = correct answers in %
